# The association between obstructive sleep apnea risk and cardiovascular disease risk in midlife Thai women in the U.S

**DOI:** 10.1007/s44470-025-00023-1

**Published:** 2026-02-05

**Authors:** Manassawee Srimoragot, Sirimon Reutrakul, Patricia E. Hershberger, Chang Park, Lauretta Quinn, Kylea Laina Liese, Bilgay Izci Balserak

**Affiliations:** 1 Department of Obstetrics and Gynecological Nursing, Faculty of Nursing, Mahidol University, Bangkok, Thailand; 2 College of Medicine, University of Illinois Chicago, Chicago, IL USA; 3 School of Nursing, University of Michigan, Ann Arbor, MI USA; 4 College of Nursing, University of Illinois Chicago, Chicago, IL USA; 5 Department of Biobehavioral Nursing Science & Center for Sleep and Health Research, College of Nursing, University of Illinois, 845 S. Damen Ave., MC 802, Chicago, IL 60612 USA

**Keywords:** Cardiovascular disease risk factors, Sleep quality, Obstructive sleep apnea, Menopause, Acculturation and asian american women

## Abstract

**Objectives:**

This study examined associations between self-reported sleep characteristics (quality, efficiency, and obstructive sleep apnea [OSA] risk), sociodemographic and cultural factors, and calculated cardiovascular disease (CVD) risk among Thai women across menopausal stages in the United States. We also evaluated whether sleep characteristics mediated or moderated these relationships.

**Methods:**

In this cross-sectional study, participants completed questionnaires assessing sleep, OSA risk, and sociodemographic factors. Weight, height, and blood pressure were measured to calculate 10-year CVD risk. Analyses included bivariate correlations, robust regression, and structural equation modeling to test mediation and moderation.

**Results:**

Among 120 participants (mean [SD] age 51.53 years [7.73]; mean calculated-CVD risk score 6.56 [5.74]), 17% had high calculated CVD risk. Length of U.S. stay was positively associated with CVD risk (*r* = 0.40, *p* < 0.001), whereas education and number of children were negatively associated (*r*=–0.22, *p* = 0.016; *r*=–0.32, *p* < 0.001). OSA risk was independently associated with higher CVD risk (B = 5.514, *p* < 0.001). Self-reported sleep quality, sleep efficiency, and OSA risk partially mediated the effects of length of stay in the U.S., number of children, employment, and anxiety on CVD risk. Sleep quality moderated the effects of education and employment on CVD risk, while OSA risk moderated the effects of education, anxiety, and menopausal symptoms.

**Conclusions:**

Self-reported OSA risk was significantly associated with higher calculated CVD risk. These findings suggest that subjective sleep disturbances, particularly OSA risk, may contribute to cardiovascular vulnerability among midlife Thai immigrant women. Longitudinal studies using objective sleep measures are needed to confirm these associations and clarify underlying mechanisms.

**Supplementary Information:**

The online version contains supplementary material available at 10.1007/s44470-025-00023-1.

## Brief Summary

**Current knowledge/study rationale:**

CVD remains a leading cause of death among women, and sleep disturbances, especially OSA, are recognized as significant modifiable risk factors. Limited research examined these associations in Asian immigrant women. We evaluated how self-reported sleep characteristics, sociodemographic, and cultural factors relate to calculated-CVD risk among Thai women residing in the U.S. across menopausal stages.

**Study impact:**

Self-reported OSA risk significantly predicted calculated-CVD risk and acted as a mediator and a moderator. Sleep quality and efficiency also contributed to these relationships. Findings support routine sleep assessments and culturally tailored interventions to improve cardiovascular health among midlife immigrant women. Causal inferences are limited by the cross-sectional design and self-reported data.

## Introduction

Sleep disturbances are highly prevalent during menopause, affecting ~ 60% of women [1], and are linked to increased cardiovascular disease (CVD) risk. Hormonal fluctuations, particularly declining estrogen, contribute to sleep and vasomotor symptoms (e.g., hot flashes) that may elevate CVD risk [2–6]. CVD remains the leading cause of death among women globally, and sleep disturbances, such as insomnia, obstructive sleep apnea (OSA), and poor sleep efficiency, are recognized as significant modifiable risk factors [7, 8]. Chronic insomnia with short sleep duration (≤ 5 h) has been independently associated with elevated risk of hypertension and other CVD events in women [8, 9]. Similarly, poor sleep during menopause, characterized by difficulty falling asleep and frequent awakenings, has been linked to higher coronary heart disease [3, 5, 9–11]. Low sleep efficiency further contributes to increased risks of CVD mortality, heart failure, myocardial infarction, and stroke [12]. OSA also significantly increases CVD risk among midlife women [13].

Most U.S.-based research on CVD and sleep has focused on White women, with limited inclusion of Asian immigrant populations [8]. Acculturation stress, cultural adaptation, and length of U.S. residency may influence sleep and CVD risk among immigrants [14–17]. However, few studies have examined these relationships among Thai women, particularly using self-reported sleep and CVD risk measures. Extreme sleep durations are more common in low-income and racial/ethnic minority populations [18]. Asians are two to three times more likely than Whites to report very short sleep (< 5 h) [19] and have higher mortality from myocardial infarction, angina, congestive heart failure, and stroke [20]. Although CVD risk among menopausal women in Thailand is high (73.8%) [21], no studies have explored how acculturation and sleep characteristics influence CVD risk in Thai women residing in the U.S, particularly those undergoing the menopausal transition. Acculturative stress may further heighten vulnerability to both sleep disturbances and CVD.

Therefore, this study aimed to examine relationships between sleep characteristics (quality, efficiency, OSA risk), socio-demographic and cultural factors, and CVD risk in Thai women across menopausal stages living in the USA. Specifically, we investigated whether sleep characteristics mediate or moderate the associations between demographic, sociocultural, and health-related factors and CVD risk. We hypothesized that self-reported sleep disturbances (poor sleep quality, low sleep efficiency, and high OSA risk) would be associated with higher CVD risk and may mediate or moderate the effects of sociodemographic and health-related factors on CVD risk.

## Methods

### Study design and sample

A total of 130 women expressed interest in the study between May and November 2021 following outreach efforts through Thai community centers, churches, temples, and social media in Illinois, USA. Of these, 125 underwent eligibility screening. Inclusion criteria were: (1) self-identification as a Thai woman, (2) age 40–65 years, and (3) sufficient English proficiency to complete study questionnaires. Exclusion criteria were: (1) diagnosis of serious mental health conditions (e.g., major depressive disorder) (*n* = 0), (2) serious medical conditions (e.g., cancer) (*n* = 0), (3) history of cardiovascular disease (e.g., stroke, myocardial infarction, heart failure) (*n* = 0), (4) current use of sleep medications or supplements (to ensure observed associations reflect natural sleep patterns) (*n* = 0), (5) heavy alcohol consumption (> 3 drinks/day) or substance abuse (*n* = 0), and (6) current pregnancy or lactation (*n* = 0). Five individuals were excluded during screening: two exceeded the age limit (> 65 years), and three lacked sufficient English proficiency. No participants were excluded based on medical or behavioral criteria. Thus, 125 women were deemed eligible, and 120 completed the full study protocol, forming the final analytic sample.

A priori sample size estimation was conducted in consultation with our statistician. Using G*Power software (version 3.1.9.4), we calculated that a minimum of 113 participants would be required to detect a medium effect size (f² = 0.26) for the primary association between OSA risk and CVD risk, assuming 80% power and a two-tailed α = 0.05. The expected effect size was informed by prior research in postmenopausal Asian women [22]. Our final analytic sample of 120 participants exceeded this requirement, providing adequate power for our primary analyses.

## Procedures

This study was approved by the Institutional Review Board of the University of Illinois Chicago (protocol #2020 − 0738). Participants provided written informed consent. Participants completed self-report questionnaires on demographic, socio-cultural, and health-related characteristics, as well as sleep parameters and cardiovascular risk. Study procedures were based on a previously published protocol [23]. After the survey, anthropometric and blood pressure measurements were obtained by a trained investigator.

## Measurements

All sleep-related measures were self-reported using validated instruments (e.g., PSQI, Berlin Questionnaire). CVD risk was estimated incorporating self-reported and measured variables (e.g., blood pressure, Body Mass Index [BMI]).

**Anthropometric measures:** Height was measured to the nearest 0.1 cm using a portable stadiometer (Seca model 217: Seca, Chino, CA), and weight to the nearest 0.01 kg using a digital scale (CAS model PB: CAS - USA Corp, East Rutherford, NJ) [24]. During the measurements, the participants were asked to wear light clothing and no shoes. Each measurement was taken twice by the investigator (MS), and a third was obtained if the two values differed by more than 0.5 units. The mean of the two closest values was used for analysis. BMI was calculated as weight (kg)/height^2^ (m^2^) [25]. 

**Blood pressure** was measured using a calibrated sphygmomanometer (BP7250: Omron Healthcare Inc., 2021) [26] to the nearest 2 mmHg in a sitting position five minutes apart on three separate occasions. The participants were asked to be seated, legs uncrossed, feet flat on the floor for five minutes, and refrain from talking while the BP was measured. The average of three readings was used for the analysis.

**Calculated cardiovascular risk:** Framingham Risk Score using BMI (FRS-BMI) was used to calculate estimated 10-year risk of CVD [27] rather than directly measure clinical outcomes. The algorithm includes age, gender, systolic blood pressure, antihypertensive use, history of diabetes, smoking status, and BMI. The FRS-BMI was validated in a worldwide culturally diverse population, including Asians [28]. It demonstrated good discriminant validity (c statistics − 0.79 in women) [27]. The FRS-BMI demonstrates moderate agreement with the original lipid -based score and reported to have moderate agreement with kappa values at 0.51 and 0.50 in women aged < 60 years and 60–74 years, respectively [29]. Composite risk scores were computed using the calculator and categorized [27] as low (< 10%), moderate (10–20%), or high (≥ 20%) [29]. The 10-year CVD risk for women can also be calculated as 1-0.948^exp(ΣßX – 26.0145)^ where ß is the regression coefficient and X is the level for each risk factor [27]. The test-retest reliability for Thai women in this study was 0.95, indicating good reliability.

**Acculturation** was assessed with the Suinn-Lew Asian Self-Identity Acculturation (SL-ASIA) [30, 31]. The SL-ASIA is a validated tool widely used among Asian population [31]. Scores range from 1 to 5 with a higher score indicating a high level of acculturation (i.e., more Americanized identity) [30].

**Pittsburgh Sleep Quality Index (PSQI)** consists of 19 self-rated questions [32]. It was used to measure habitual sleep quality and quantity over the previous month. The PSQI is composed of 7 subscales assessing subjective sleep quality, sleep latency, sleep duration, habitual sleep efficiency, sleep disturbances, use of sleep medication, and daytime dysfunction. Each subscale has a possible score between 0 and 3, with an overall global score of 0–21 with higher scores indicating poor sleep quality. Participants with a score of ≥ 5 were considered as poor sleepers. Sleep efficiency was calculated from the habitual sleep efficiency subscale with a formula of (hours of sleep/hours in bed)*100. It was reported as a percentage with higher scores indicating higher sleep efficiency [32].

**Berlin questionnaire** is a widely used, self-administered screening tool to identify individuals at high risk of having OSA [33]. It consists of 10 questions on the following components: snoring, tiredness, observed apnea, and high blood pressure classified into three categories: (1) the presence and severity of snoring, (2) frequency of daytime sleepiness, and (3) the presence of obesity or hypertension. The questionnaire has been used and confirmed to be valid among premenopausal and menopausal women [34]. The sensitivity and specificity range from 57 to 71% and 86–96%, respectively. A positive score for two or more categories indicates a high likelihood of high OSA risk [35].

**Menopausal status** is classified following the stage of Reproductive Aging Workshop (STRAW) criteria [36]. This defines reproductive stages or menstrual period in the past 12 months and reason for irregular/no periods into 3 stages: (1) Premenopause (regular period), (2) Perimenopause (irregular period or changes in menstrual cycles), and (3) Post menopause (no period in the past 12 months). These clinical criteria provide standardized staging widely used in menopausal research.

**Menopausal symptoms** were measured using the Menopause Rating Scale (MRS) [37]. The MRS consisted of 11 questions with 3 dimensions (psychological, somato-vegetative, and urogenital symptoms) and 5 rating scales (0–4). The total MRS ranges from 0 to 44 which corresponds with the symptoms from asymptomatic to the highest degree of complaints. The severity of menopausal symptoms can be divided into 3 categories: (1) 5–8 = mild; (2) 9–15 = moderate; and 3) > 15 = severe.

**Anxiety** was evaluated using the Patient-Reported Outcomes Measurement Information System – Short Form version 1.0 (PROMIS-SF v1.0) – Anxiety 4a [38]. It measured anxiety scores based on 4 questions regarding fearfulness, difficulty focusing, feeling overwhelmed, and uneasiness. Each item has 5 scales (never, rarely, sometimes, often, and very often). The score was auto-calculated into t-score, with higher t-scores indicating a higher level of anxiety.

**Covariates and determinants:** These included length of U.S. residence, education, household income, marital status, number of children, employment, acculturation, anxiety, menopausal symptoms, alcohol consumption, exercise, sleep quality, sleep efficiency, and OSA risk. Selection was based on theoretical relevance and/or significant associations observed in preliminary analyses.

### Statistical analyses

Data were analyzed using STATA 17.1. Descriptive statistics summarized participant characteristics, sleep variables, and cardiovascular risk indicators. Model assumptions (linearity, normality, homoscedasticity, and multicollinearity) were assessed and addressed where necessary. Spearman’s rho was used for bivariate analyses due to non-normality. Robust regression was applied to address right-skewness of the CVD risk score and to mitigate the influence of outliers and heteroscedasticity [39], while acknowledging that the cross-sectional, self-reported nature of the data limits causal inference.

Variables with *p* < 0.20 in bivariate analyses were retained in multivariable models to minimize Type II errors. Demographic and health factors (e.g., length of U.S. residence, education, income, number of children, employment, anxiety, menopausal symptoms) were included. Marital status, acculturation, alcohol use, and exercise were added to sleep models due to their associations with sleep variables. Sleep quality, sleep efficiency, and OSA risk were examined as potential mediators or moderators of CVD risk. Smoking status was excluded due to its very low prevalence (1.67%), consistent with WHO data on Thai women [40], as its inclusion caused unstable estimates and wide confidence intervals.

#### Mediation and moderation analysis

Structural Equation Modeling (SEM) was conducted to evaluate mediating pathways [41, 42] using a path model based on correlation matrices. Indirect effects were tested using product-of-coefficients methods and were considered significant at *p* < 0.05. Moderation analyses using interaction terms assessed whether sleep variables (quality, efficiency, and OSA risk) modified the associations between socio-demographic or health-related variables and CVD risk. Statistical significance for all tests was set at *p* < 0.05.

## Results

### Participant characteristics

Participant characteristics are summarized in Table 1. The mean age was 51.53 years (SD 7.73), with an average duration of residence in the U.S. of 21.77 years (12.96). Most participants were foreign-born (96.7%), college-educated (77.5%), and reported annual incomes greater than $70,000 (55.8%). The majority were married (75.0%), had one to two children (88.3%), and were employed (71.0%), with 12.5% working night shifts. The mean acculturation score was 1.90 (0.58), indicating low-to-moderate acculturation.

**Table 1:** Demographic, acculturation, sleep-, and health-related characteristics of participants

Variables	Results		
	Mean ± SD	N (percent)	Range: min-max
Age, years	51.53±7.73		40.00–65.00
Length of stay in the USA, years	21.77±12.96		1.00–52.00
Birthplace, n (%)			
Outside US		116 (96.67%)	
US		4 (3.33%)	
Education, n (%)			
High school equivalent or less		12 (10.00%)	
Some college or associate degree		15 (12.50%)	
College degree or higher		93 (77.50%)	
Household income/year, USD, n (%)			
< $0 - $30,000		12 (10.00%)	
$30,001–70,000		41 (34.17%)	
> $70,000		67 (55.83%)	
Marital status, n (%)			
Single		19 (15.83%)	
Married/partnered		90 (75.00%)	
Separated/Widowed		11 (9.17%)	
Numbers of children, n (%)			
None		106(88.33%)	
1–2 children		14 (11.67%)	
Employment status, n (%)			
Unemployed		35 (29.17%)	
Employed		85 (70.83%)	
Night shift work, n (%)		15 (12.50%)	
Acculturation, SL-ASIA	1.90 ± 0.58	0.59	1.00–4.00
CVD risk: FRS-BMI	6.56 ± 5.74		1.00–30.00
CVD risk category, n (%)			
Low		100 (83.33%)	
Moderate		16 (13.33%)	
High		4 (3.33%)	
Sleep quality, PSQI	4.18 ± 3.03		0.00–17.00
Sleep quality status, n (%)			
Good		97 (80.83%)	
Poor		23 (19.17%)	
Sleep efficiency, PSQI, (%)	95.36 ± 5.96		63.64–100.00
OSA risk: Berlin questionnaire, n (%)			
Low		103 (85.83%)	
High		17 (14.17%)	
Anxiety, PROMIS-SF v1.0	44.60 ± 7.31		40.30–69.50
Menopause symptoms, MRS	63 ± 4.71		0.00–23.00
Menopause symptoms, n (%)			
None		87 (72.50%)	
Mild		18 (15.00%)	
Moderate		10 (8.33%)	
Severe		5 (4.17%)	
Menopause status, n (%)			
Premenopause		38 (31.67%)	
Perimenopause		14 (11.67%)	
Postmenopause		68 (56.67%)	
Smoking status, n (%)			
Never		117 (97.50%)	
Former		1 (0.83%)	
Current		2 (1.67%)	
Alcohol consumption, n (%)			
Never/monthly or less		112 (93.33%)	
Occasionally (2–4 times/month)		4 (3.33%)	
Sometimes 2–3 times/wk		3 (2.50%)	
Often (≥ 4/wk)		1 (0.83%)	
Exercise time, n (%)			
< 30 min per day		37 (30.83%)	
30–60 min per day		71 (59.17%)	
> 60 min per day		12 (10.00%)	
Chronic disease diagnosis, n (%)			
No		92 (76.67%)	
Yes		28 (23.33%)	
BMI, kg/m^2^	24.17 ± 4.01		16.56–39.63
Weight, kg	59.30 ± 11.66		37.27–98.93
Height, cm	156.51 ± 5.86		141.00–168.00
Systolic BP, mmHg	125.35 ± 20.7		90.00–193.00
Diastolic BP, mmHg	79.09 ± 12.72		52.00–122.00

Results are shown in mean ± standard deviation; *SL-ASIA,* Suinn-Lew Asian Self-Identity Acculturation; *CVD,* cardiovascular disease; *FRS-BMI,* Framingham risk score-BMI based; *PSQI,* Pittsburgh Sleep Quality Index; *OSA,* obstructive sleep apnea; *MRS,* Menopausal Rating Scale; *BMI,* body mass index; *BP,* blood pressure

The mean calculated CVD risk score (FRS–BMI) was 6.56 (5.74), ranging from 1% to 30%. Most participants (83.3%) were categorized as low risk (< 10%), and 17% as high risk (≥ 20%). All sleep characteristics, including sleep quality, sleep efficiency, and risk for OSA, were derived from participant self-reported questionnaires. The mean PSQI score was 4.18 (3.03); 80.8% were classified as good sleepers. Self-reported sleep efficiency averaged 95.36% (5.96), and 14.2% screened positive for high self-reported OSA risk.

Participants had a mean anxiety T-score of 44.60 (7.31). Menopausal symptoms averaged 3.63 (4.71); 72.5% reported no symptoms and 15.0% mild symptoms. Proportions of premenopausal, perimenopausal, and postmenopausal participants were 31.7%, 11.7%, and 56.7%, respectively. Most participants were non-smokers (97.5%) and infrequent alcohol consumers (93.3%). 59% exercised 30–60 min daily. Twenty-eight participants had at least one chronic disease. The mean BMI was 24.17 kg/m² (4.01), mean systolic BP was 125.35 mmHg (20.72), and mean diastolic BP was 79.09 mmHg (12.72).

### Associations of sociodemographic, health, and sleep variables with calculated cardiovascular disease risk

In bivariate correlations (Table 2), length of stay in the U.S. was positively correlated with CVD risk (*r* = 0.404, *p* < 0.001), whereas education (*r*=–0.220, *p* = 0.016) and number of children (*r*=–0.322, *p* < 0.001) were negatively correlated. Variables with p-values < 0.20, household income (*r*=–0.138), employment (*r*=–0.146), anxiety (*r* = 0.174), and menopausal symptoms (*r* = 0.120), were retained for multivariate modeling. Variables such as marital status, night shift work, acculturation, smoking, alcohol consumption, and exercise time were excluded due to limited variability (Fig. S1). Birthplace and smoking status were also excluded from the bivariate analysis due to high variability.

**Table 2:** Bivariate associations among CVD risk, sleep characteristics, and participants characteristics

Variables	CVD risk ^a^	Sleep quality ^a^	Sleep efficiency ^a^	Risk of OSA ^a^
*Demographics*				
Age, years	0.782***	0.065	−0.013	0.084
*p*	0.000	0.479	0.891	0.362
Length of stay in the USA, years	0.404***	−0.129^†^	0.057	0.054
*p*	0.000	0.160	0.538	0.559
Education, n (%)	−0.220*	−0.026	0.132^†^	0.051
*p*	0.016	0.775	0.149	0.579
Household income per year, USD, n (%)	−0.138^†^	−0.179^†^	0.128^†^	0.099
*p*	0.133	0.051	0.165	0.285
Marital status, n (%)	−0.018	0.142^†^	−0.163^†^	0.108
*p*	0.847	0.122	0.076	0.241
Numbers of children, n (%)	−0.322***	−0.020	−0.087	−0.148^†^
*p*	0.000	0.831	0.343	0.108
Employment status, n (%)	−0.146^†^	−0.031	0.144^†^	−0.103
*p*	0.111	0.734	0.117	0.263
Night shift work, n (%)	0.015	0.081	−0.079	−0.081
*p*	0.875	0.381	0.393	0.377
Acculturation, SL-ASIA	0.036	−0.157^†^	0.171^†^	−0.073
*p*	0.699	0.086	0.062	0.432
*Health-related factors*				
BMI, kg/m^2^	0.426***	−0.011	0.048	0.226*
*p*	0.000	0.908	0.601	0.013
Anxiety, PROMIS-SF v1.0	0.174^†^	0.281**	−0.152^†^	0.112
*p*	0.057	0.002	0.097	0.224
Menopause symptoms, MRS	0.120^†^	0.454***	−0.318***	0.303***
*p*	0.190	0.000	0.000	0.001
Smoking status, n (%)	0.048	−0.050	−0.004	−0.065
*p*	0.602	0.588	0.967	0.480
Alcohol consumption, n (%)	−0.081	−0.150^†^	0.079	−0.109
*p*	0.380	0.103	0.390	0.238
Exercise time, n (%)	−0.045	−0.059	−0.057	−0.226*
*p*	0.629	0.525	0.537	0.013
Chronic disease diagnosis, n (%)	0.440***	0.233*	−0.224*	0.454***
*p*	0.000	0.011	0.014	0.000
Average systolic BP	0.853***	−0.078	0.162^†^	0.368***
*p*	0.000	0.397	0.076	0.000
Average diastolic BP	0.510***	−0.139^†^	0.231*	0.233*
*p*	0.000	0.130	0.011	0.011

*p*, p value; ^†^ *p* < 0.20, *p < 0.05, **p < 0.01, ***p < 0.001; *CVD*, cardiovascular disease; *OSA*, obstructive sleep apnea; *SL-ASIA*, Suinn-Lew Asian Self-Identity Acculturation; *BMI*, body mass index; *MRS*, Menopausal Rating Scale; *BP*, blood pressure

^a^ Non-parametric correlation: Spearman rho

Self-reported sleep quality was significantly correlated with anxiety (*r* = 0.281, *p* = 0.002), menopausal symptoms (*r* = 0.454, *p* < 0.001), and chronic disease (*r* = 0.233, *p* = 0.011). Additional variables associated at *p* < 0.20 included length of stay in the U.S. (*r*=–0.129), household income (*r*=–0.179), acculturation (*r*=–0.157), alcohol use (*r*=–0.159), and diastolic BP (*r*=–0.139) (Table 2).

Sleep efficiency was negatively correlated with menopausal symptoms (*r*=–0.318, *p* < 0.001) and chronic disease (*r*=–0.224, *p* = 0.014), and positively correlated with diastolic BP (*r* = 0.231, *p* = 0.011). Other variables associated at *p* < 0.20 were education (*r* = 0.132), income (*r* = 0.128), marital status (*r*=–0.163), employment (*r* = 0.144), acculturation (*r* = 0.171), anxiety (*r*=–0.152), and systolic BP (*r* = 0.162).

OSA risk was significantly associated with BMI (*r* = 0.226, *p* = 0.013), menopausal symptoms (*r* = 0.303, *p* = 0.001), chronic disease (*r* = 0.454, *p* < 0.001), exercise time (*r*=–0.226, *p* = 0.013), systolic BP (*r* = 0.368, *p* < 0.001), and diastolic BP (*r* = 0.233, *p* = 0.011). Number of children (*r*=–0.148) was the only variable associated at *p* < 0.20.

**Fig. 1:**
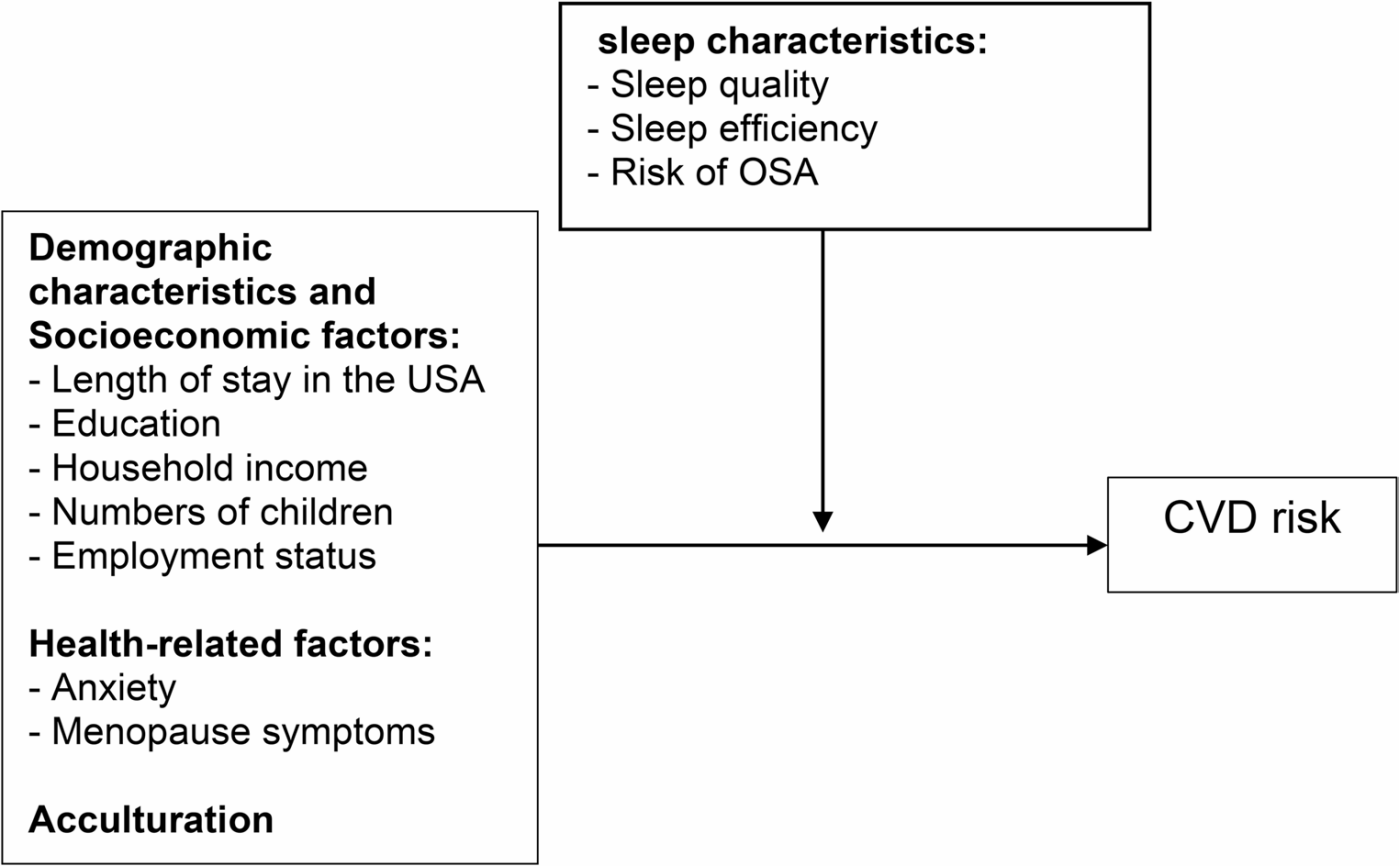
Proposed model to estimate paths toward cardiovascular disease risk (moderation). *CVD*, cardiovascular disease; *OSA*, obstructive sleep apnea

Variables used to compute the FRS-BMI (BMI, chronic disease, systolic and diastolic BP) were excluded from CVD regression analyses to avoid multicollinearity. The final CVD risk models were adjusted for length of stay in the U.S., education, income, number of children, employment, anxiety, and menopausal symptoms, which showed direct associations with CVD risk. Marital status, acculturation, alcohol use, and exercise time were tested as indirect predictors through sleep-related variables but were not included in mediation models (Fig. 1; Fig. S2).

### Self-reported sleep characteristics as predictors of CVD risk (FRS-BMI)

In unadjusted robust regression models, only OSA risk was significantly associated with higher CVD risk (B = 4.601, *p* < 0.001). The association remained significant in the regression model after adjusting for covariates including length of stay in the U.S., education level, income, anxiety, menopausal symptoms, acculturation, alcohol use, and exercise (B = 5.514, *p* < 0.001; Table S1). Self-reported sleep quality and sleep efficiency were not significant predictors of CVD risk.

### Mediation analysis

Robust regression analysis (Table 3) showed that length of stay in the U.S. (B = 0.112, *p* < 0.001), education (college or higher; B=–3.191, *p* = 0.007), number of children (1–2 children; B=–2.963, *p* = 0.006), and menopausal symptoms (B=–0.152, *p* = 0.050) were directly associated with CVD risk.

**Table 3:** Summary of robust regression of participants characteristics, socioeconomic factors, health-related factors, and CVD risk

Independent variables	Dependent variables					
	CVD risk ^a^					
	Coefficient	SE	t	*p*	95% CI	
Length of stay in the USA, years	0.112	0.028	4.050	0.000	0.057	0.167
Education, n (%)						
High school equivalent or less						
Some college or associate degree	−2.884	1.407	−2.050	0.043	−5.676	−0.093
College degree or higher	−3.191	1.160	−2.750	0.007	−5.493	−0.890
Household income per year, USD, n (%)						
< $0 - $30,000						
$30,001–70,000	−0.259	1.208	−0.210	0.830	−2.655	2.136
> $70,000	−1.566	1.215	−1.290	0.200	−3.975	0.843
Numbers of children, n (%)						
None						
1–2 children	−2.963	1.051	−2.820	0.006	−5.049	−0.878
Employment status, n (%)						
Unemployed						
Employed	−1.430	0.780	−1.830	0.070	−2.977	0.117
Anxiety, PROMIS-SF v1.0	0.082	0.049	1.670	0.098	−0.016	0.180
Menopause symptoms, MRS	−0.152	0.077	−1.980	0.050	−0.304	0.000
Marital status, n (%)						
Single						
Married/partnered	0.534	0.998	0.540	0.593	−1.445	2.514
Separated/Widowed	0.784	1.487	0.530	0.599	−2.167	3.734
Acculturation, SL-ASIA	−0.667	0.655	−1.020	0.310	−1.966	0.631
Alcohol consumption, n (%)						
Never/monthly or less						
Occasionally (2–4 times/month)	−0.107	1.835	−0.060	0.954	−3.746	3.532
Sometimes 2–3 times/wk	−1.545	2.208	−0.700	0.486	−5.924	2.834
Often (≥ 4/wk)	2.971	3.666	0.810	0.420	−4.300	10.243
Exercise time, n (%)						
< 30 min						
30–60 min	−0.092	0.754	−0.120	0.903	−1.587	1.403
> 60 min	0.835	1.304	0.640	0.523	−1.750	3.421
	F(17, 102) = 3.71, *p* = 0.000					

*SE*, standard error; *t*, t-test; *p*, p value; *95% CI*, 95% confidence interval; *CVD*, cardiovascular disease; *MRS*, Menopausal Rating Scale; *SL-ASIA*, Suinn-Lew Asian Self-Identity Acculturation; ^a^ Robust regression

SEM further examined whether self-reported sleep characteristics mediated these relationships (Table 4). Sleep quality partially mediated the associations of length of stay in the U.S. (B = 0.114, *p* = 0.001), number of children (B=–3.175, *p* = 0.025), employment (B=–2.893, *p* = 0.004), and anxiety (B = 0.240, *p* < 0.001) with CVD risk. Similarly, sleep efficiency mediated the effects of length of stay in the U.S. (B = 0.117, *p* = 0.001), number of children (B=–3.102, *p* = 0.029), employment (B=–2.899, *p* = 0.004), and anxiety (B = 0.234, *p* < 0.001) on CVD risk. Concurrently, OSA risk significantly mediated associations of length of stay in the U.S. (B = 0.115, *p* < 0.001), employment (B=–3.098, *p* = 0.001), and anxiety (B = 0.238, *p* < 0.001) with CVD risk. These variables were incorporated into a multiplicative mediation model.

**Table 4:** Summary of structural equation model of participants characteristics, socioeconomic factors, health-related factors, sleep characteristics (mediator), and CVD risk

	Dependent variables: CVD risk											
Total effect on CVD risk (Independent variables)	Mediator ^a^											
	Sleep quality ^a^				Sleep efficiency ^a^				Risk of OSA ^a^			
	Coef.	*p*	95% CI		Coef.	*p*	95% CI		Coef.	*p*	95% CI	
Length of stay in the USA, years	0.114	0.001	0.046	0.181	0.117	0.001	0.050	0.184	0.115	0.000	0.054	0.177
Education, n (%)												
High school equivalent or less												
Some college or associate degree	−0.829	0.664	−4.565	2.908	−0.867	0.653	−4.643	2.910	−0.848	0.628	−4.280	2.584
College degree or higher	−2.180	0.159	−5.211	0.851	−2.225	0.156	−5.299	0.849	−2.274	0.109	−5.058	0.510
Household income per year, USD, n (%)												
< $0 - $30,000												
$30,001–70,000	2.109	0.205	−1.155	5.373	2.082	0.212	−1.190	5.354	0.923	0.551	−2.113	3.958
> $70,000	−0.444	0.781	−3.578	2.689	−0.469	0.770	−3.609	2.671	−1.766	0.237	−4.693	1.161
Numbers of children, n (%)												
None												
1–2 children	−3.175	0.025	−5.954	−0.397	−3.102	0.029	−5.887	−0.316	−2.296	0.080	−4.867	0.275
Employment status, n (%)												
Unemployed												
Employed	−2.893	0.004	−4.862	−0.925	−2.899	0.004	−4.884	−0.914	−3.098	0.001	−4.908	−1.289
Anxiety, PROMIS-SF v1.0	0.240	0.000	0.110	0.369	0.234	0.000	0.104	0.365	0.238	0.000	0.119	0.356
Menopause symptoms, MRS	0.167	0.166	−0.069	0.403	0.130	0.252	−0.092	0.352	−0.036	0.719	−0.231	0.159
Mediator ^a^	−0.136	0.444	−0.484	0.212	0.020	0.813	−0.142	0.182	5.896	0.000	3.483	8.309
Direct effect with mediators (Independent variable)	Mediator ^a^											
	Sleep quality ^a^				Sleep efficiency ^a^				Risk of OSA ^a^			
	Coef.	*p*	95% CI		Coef.	*p*	95% CI		Coef.	*p*	95% CI	
Length of stay in the USA, years	−0.020	0.288	−0.056	0.017	0.001	0.981	−0.077	0.079	0.003	0.303	−0.002	0.007
Education, n (%)												
High school equivalent or less												
Some college or associate degree	−0.618	0.511	−2.459	1.224	4.334	0.033	0.355	8.312	0.045	0.724	−0.203	0.292
College degree or higher	0.140	0.857	−1.379	1.658	2.887	0.084	−0.393	6.168	0.074	0.475	−0.130	0.278
Household income per year, USD, n (%)												
< $0 - $30,000												
$30,001–70,000	0.349	0.665	−1.231	1.930	−0.057	0.974	−3.471	3.358	0.182	0.093	−0.030	0.394
> $70,000	0.815	0.315	−0.775	2.404	−1.040	0.553	−4.474	2.394	0.185	0.089	−0.028	0.399
Numbers of children, n (%)												
None												
1–2 children	−0.632	0.368	−2.009	0.744	−0.775	0.609	−3.748	2.197	−0.150	0.112	−0.335	0.035
Employment status, n (%)												
Unemployed												
Employed	−0.266	0.609	−1.287	0.755	1.217	0.280	−0.988	3.422	0.042	0.552	−0.095	0.179
Anxiety, PROMIS-SF v1.0	0.034	0.297	−0.030	0.099	0.073	0.306	−0.067	0.212	−0.002	0.652	−0.011	0.007
Menopause symptoms, MRS	0.364	0.000	0.264	0.464	−0.598	0.000	−0.814	−0.381	0.025	0.000	0.012	0.039
Marital status, n (%)												
Single												
Married/partnered	−0.745	0.264	−2.051	0.562	1.661	0.248	−1.160	4.482	0.200	0.025	0.025	0.376
Separated/Widowed	0.257	0.796	−1.690	2.203	−1.094	0.610	−5.299	3.111	0.120	0.368	−0.141	0.382
Acculturation, SL-ASIA	−1.043	0.017	−1.900	−0.187	2.526	0.007	0.676	4.377	−0.038	0.518	−0.153	0.077
Alcohol consumption, n (%)												
Never/monthly or less												
Occasionally (2–4 times/month)	−1.483	0.226	−3.884	0.918	0.996	0.707	−4.190	6.182	−0.020	0.903	−0.342	0.302
Sometimes 2–3 times/wk	1.424	0.334	−1.466	4.313	−0.765	0.810	−7.005	5.476	0.037	0.853	−0.351	0.425
Often (≥ 4/wk)	−6.365	0.009	−11.163	−1.567	8.827	0.095	−1.537	19.191	−0.324	0.324	−0.969	0.320
Exercise time, n (%)												
< 30 min												
30–60 min	0.137	0.785	−0.849	1.124	−1.922	0.077	−4.052	0.209	−0.153	0.024	−0.285	−0.021
> 60 min	1.371	0.115	−0.335	3.077	−4.020	0.033	−7.705	−0.334	−0.142	0.226	−0.371	0.088

*Coef*, Coefficient; *p*, p value; *95% CI*, 95% confidence interval; *CVD*, cardiovascular disease; *OSA*, obstructive sleep apnea; *MRS*, Menopausal Rating Scale;* SL-ASIA*, Suinn-Lew Asian Self-Identity Acculturation

**Fig. 2:**
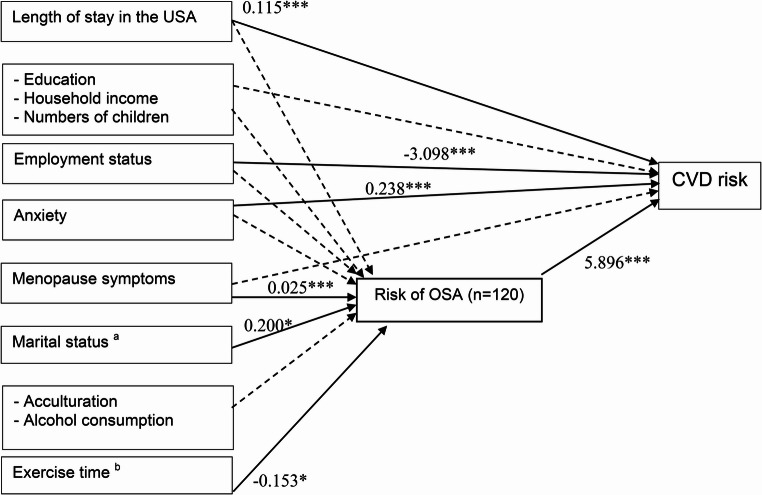
Path diagram for the final mediation model of risk of obstructive sleep apnea. Non-standardized estimates are reported for statistically significant effects shown as solid lines Dash line represent paths that were estimated but not statistically significant. The indirect effect of menopause symptoms was significant (coefficient = 0.149, *p* = 0.004. Other covariates: length of stay in the USA, marital status, and exercise time did not show indirect effect with CVD risk. *CVD*, cardiovascular disease; *OSA*, obstructive sleep apnea; * *p* < 0.05, ** *p* < 0.01, *** *p* < 0.001; ^a^ only in single group; ^b^ only if exercise time was 30–60 min per day

Subgroup analyses among women with children revealed a direct effect of having 1–2 children on CVD risk (Table S2) without indirect mediation via sleep pathways. However, menopausal symptoms had indirect effects on CVD risk mediated through elevated OSA risk (B = 0.149, *p* = 0.004; Fig. 2). A comprehensive summary of direct and indirect effects is reported in Table S3. While length of stay in the U.S., number of children, employment, and anxiety had direct associations with CVD risk, these were not mediated by sleep quality or efficiency. In contrast, OSA risk emerged as a key mediator linking menopausal symptoms to increased CVD risk.

### Moderation analysis

Interaction terms to determine whether sleep characteristics modified the associations between demographic and health-related factors and CVD risk (Table S4). Sleep quality emerged as a significant moderator of the relationship between education level and employment status and CVD risk. Specifically, the interaction between sleep quality and education was significant for participants with some college or an associate degree (B = 0.888, *p* = 0.032) and for those with a college degree or higher (B = 1.069, *p* = 0.001). Similarly, the interaction between sleep quality and employment status was significantly associated with CVD risk (B = 0.648, *p* = 0.005) (Fig. S3). The risk of OSA also significantly moderated the associations of education (college degree or higher, B = 8.493, *p* < 0.001), anxiety (B = 0.878, *p* < 0.001), and menopausal symptoms (B = 0.772, *p* < 0.001) with CVD risk (Fig. S3). No significant moderating effects were observed for sleep efficiency.

Other covariates including length of stay in the USA, household income, number of children, employment status, and acculturation were statistically controlled in all models. However, their interaction terms with sleep-related variables were not significant and are therefore not discussed further (Table S4). Table S5 summarizes the moderation analyses, illustrating how sleep characteristics interact with key sociodemographic and health factors to influence CVD risk.

In summary, self-reported sleep quality significantly moderated the effects of education and employment status on CVD risk, while OSA risk moderated relationships involving anxiety and menopausal symptoms. These interactions highlight the importance of considering sleep health not only as a direct predictor but also as a potential effect modifier in cardiovascular risk assessment among women.

## Discussion

This study examined whether self-reported sleep characteristics (sleep quality, sleep efficiency, and OSA risk) mediate or moderate relationships between demographic, sociocultural, and health-related factors and calculated CVD risk (FRS-BMI). Most participants showed favorable cardiovascular and sleep profiles, including low calculated CVD risk, good self-reported sleep quality, and high sleep efficiency. However, higher self-reported OSA risk was associated with greater calculated CVD risk. OSA risk acted as a potential mediator between menopausal symptoms and CVD risk and a moderator between anxiety, menopausal symptoms, and CVD risk. Self-reported sleep quality moderated the associations between education, employment, and calculated CVD risk. These cross-sectional findings may contribute to prior work on sleep, menopause, and CVD risk among Asian women.

The overall low CVD risk profile of our sample likely reflects relatively healthy lifestyle behaviors, consistent access to healthcare, and socioeconomic advantages. This contrasts with prior research among Thai women in Thailand, where estimated CVD risk was 73.8% [21]. Only 11.7% of our participants had children, markedly lower than national averages [43]. Within this subgroup, a lower CVD risk was observed, which may suggest a possible association between parenthood and reduced cardiovascular risk in high-resource settings; however, this finding should be interpreted as a hypothesis rather than a definitive causal link. The high socioeconomic status of our sample (70.8% employed, 55.8% earning >$70,000 annually) [44, 45] may also contribute to lower CVD risk, as delayed parenthood and economic stability could support healthier behaviors and reduced stress, indirectly benefiting cardiovascular health [46].

Length of U.S. residency was positively associated with calculated CVD risk. While dietary acculturation (e.g., adoption of Western diets) may partly explain this, modest acculturation scores (mean SL-ASIA = 1.90) suggest other mechanisms such as chronic stress or reduced physical activity may also play roles. The association between self-reported OSA risk and calculated CVD risk aligns with evidence linking sleep-disordered breathing to cardiometabolic dysfunction [47, 48]. However, the self-reported nature of these measures limits causal conclusions. Differences in sleep and CVD associations have also been observed in Asian subgroups. For example, Nadarajah et al. [49] reported that Asian Indians and Chinese had better sleep compared to Filipinos and Other Asians, but these differences did not translate to significant variations in CVD risk.

Biological mechanisms underlying OSA’s effect include intermittent hypoxia and sympathetic activation, which may promote hypertension and systemic inflammation [50, 51]. Obesity, a known CVD risk factor [52], may further exacerbate this relationship, especially in Asian populations with higher fat at lower BMIs (≥ 23 kg/m²) [53]. Our sample’s mean BMI (24.17 kg/m^2^) indicates elevated cardiometabolic risk, potentially contributing to observed associations between self-reported OSA risk and calculated CVD risk.

Self-reported sleep quality was not directly associated with CVD risk but showed indirect and moderating effects, possibly limited by a ceiling effect. These findings align with studies in Chinese women linking better self-reported sleep quality to lower CVD risk [22, 54] but differ from longitudinal work using objective measures linking poor sleep efficiency to higher CVD mortality [12].

This study’s strengths include focusing on understudied Thai immigrant women across menopausal stages, enhancing generalizability within Asian immigrant groups in the U.S. Limitations include reliance on self-reported sleep data (susceptible to recall bias), use of calculated rather than measured CVD risk, a cross-sectional design preventing causal interpretations, and a relatively socioeconomically homogeneous sample, which may limit wider generalizability.

These findings suggest self-reported sleep disturbances, particularly OSA risk, may be clinically relevant in assessing CVD risk in midlife immigrant women. Clinicians might consider screening for sleep disorders in women with menopausal symptoms or anxiety and integrating evidence-based treatments such as cognitive-behavioral therapy for insomnia or continuous positive airway pressure for OSA. Culturally tailored interventions addressing acculturative stress and lifestyle factors may improve effectiveness.

## Conclusion

This study provides novel evidence linking self-reported sleep disturbances, OSA risk, and cardiometabolic health among Thai midlife women in the U.S., an understudied immigrant population. The findings underscore the need for culturally sensitive, community-based strategies to improve sleep and reduce cardiometabolic risk. Future longitudinal studies using objective sleep assessments (e.g., actigraphy or home sleep apnea testing) are warranted to clarify causal pathways.

## Supplementary Information

Below is the link to the electronic supplementary material.

Supplementary Material 1 (DOCX 17.7 KB) 

## Data Availability

The dataset generated and analyzed during the study are not publicly available due to participant confidentiality but are available from the first/corresponding author on reasonable request.

## Abbreviations

BMI: body mass index
BP: blood pressure
CI: confidence interval
CVD: cardiovascular disease
FRS-BMI: Framingham Risk Score using BMI
MRS: menopause rating scale
OSA: obstructive sleep apnea
p: p-value
PROMIS-SF v1.0: Patient-Reported Outcomes Measurement Information System–Short Form version 1.0
PSQI: Pittsburgh Sleep Quality Index
SD: standard deviation
SEM: structural equation models
SL-ASIA: Suinn-Lew Asian Self-Identity Acculturation
STRAW: Stage of Reproductive Aging Workshop
USA: United States of America
