# Supplementary material for: The association between obstructive sleep apnea risk and cardiovascular disease risk in midlife Thai women in the U.S
Source: J Clin Sleep Med. 2026 Feb 5;22(1):26. doi: 10.1007/s44470-025-00023-1 (PMC12876511; doi:10.1007/s44470-025-00023-1)
Supplement: Supplementary file 1 — Supplementary Material 1 (DOCX 17.7 KB) [file 44470_2025_23_MOESM1_ESM.docx]

**Supplemental materials**

**Table S1** summary of unadjusted and adjusted relationships of sleep-related characteristics and cardiovascular disease risk

| Independent variables | Dependent variables: CVD risk ^a^ | | | | | |  |
| --- | --- | --- | --- | --- | --- | --- | --- |
|  | Unadjusted | | | Adjusted | | |  |
|  | B | 95% CI | *p* | B | 95% CI | *p* | |
| Sleep quality ^b^ | 0.164 | -0.181, 0.508 | 0.348 | -0.171 | -0.574, 0.233 | 0.233 | |
| Sleep efficiency ^b^ | -0.023 | \| -0.142, 0.095 \| \| --- \| | 0.695 | -0.024 | -0.149, 0.101 | 0.704 | |
| Risk of OSA ^c^ | 4.601 | \| 2.687, 6.514 \| \| --- \| | 0.000 | 5.514 | 3.444, 7.584 | 0.000 | |

*Notes*. *B,* Beta; *95% CI*, 95% Confidence Interval; *p,* p value; CVD, Cardiovascular Disease; OSA, Obstructive Sleep Apnea

^a^ Robust regression

^b^ Adjusted for length of stay in the USA, education, household income, numbers of children, employment status, anxiety, menopause symptoms, marital status, acculturation, alcohol consumption, exercise time, and acculturation

^c^ Adjusted for length of stay in the USA, education, household income, numbers of children, employment status, anxiety, menopause symptoms, and acculturation

**Table S2** summary of multiplicative approach estimation of structural equation model of participants characteristics and sleep-related characteristics on cardiovascular disease risk

| Equation (Dependent variables) | Coefficient | SE | z | *p* | 95% CI | |
| --- | --- | --- | --- | --- | --- | --- |
| Sleep quality (n=120) | | | | | | |
| Length of stay in the USA | 0.003 | 0.004 | 0.620 | 0.535 | -0.006 | 0.011 |
| Numbers of children | 0.086 | 0.147 | 0.580 | 0.560 | -0.203 | 0.374 |
| Employment status | 0.036 | 0.085 | 0.430 | 0.671 | -0.131 | 0.203 |
| Anxiety | -0.005 | 0.008 | -0.620 | 0.537 | -0.019 | 0.010 |
| Menopause symptoms | -0.049 | 0.065 | -0.760 | 0.447 | -0.177 | 0.078 |
|  |  |  |  |  |  |  |
| Sleep efficiency (n=120) | | | | | | |
| Length of stay in the USA | 0.000 | 0.001 | 0.020 | 0.981 | -0.002 | 0.002 |
| Education ^a^ | 0.085 | 0.360 | 0.230 | 0.814 | -0.622 | 0.791 |
| Numbers of children | -0.015 | 0.071 | -0.210 | 0.830 | -0.153 | 0.123 |
| Employment status | 0.024 | 0.103 | 0.230 | 0.817 | -0.178 | 0.226 |
| Anxiety | 0.001 | 0.006 | 0.230 | 0.818 | -0.011 | 0.014 |
| Menopause symptoms | -0.012 | 0.049 | -0.240 | 0.813 | -0.109 | 0.085 |
|  |  |  |  |  |  |  |
| Risk of OSA (n=120) |  |  |  |  |  |  |
| Length of stay in the USA | 0.015 | 0.015 | 1.010 | 0.314 | -0.014 | 0.044 |
| Employment status | 0.245 | 0.416 | 0.590 | 0.555 | -0.569 | 1.060 |
| Anxiety | -0.012 | 0.026 | -0.450 | 0.653 | -0.063 | 0.040 |
| Menopause symptoms | 0.149** | 0.051 | 2.910 | 0.004 | 0.049 | 0.249 |

*Notes*. *SE,* Standard Error; *z,* z- score; *p,* p value; *95% CI*, 95% Confidence Interval; *p<0.05, **p<0.01, ***p < 0.001; CVD, Cardiovascular Disease; OSA, Obstructive Sleep Apnea; ^a^ Some college or association degree

**Table S3** summary of the direct and indirect effects of participants characteristics, socioeconomic factors, health-related factors, and sleep-related characteristics on cardiovascular disease risk

| Independent variables | Dependent variables | | |
| --- | --- | --- | --- |
|  | CVD risk (n=120) | | |
|  | Direct effect | Indirect effect | Mediator |
| Length of stay in the USA, years | Yes ^b^ | No | No |
| Education, n (%) | No | No | No |
| Household income per year, USD, n (%) | No | No | No |
| Numbers of children, n (%) | Yes ^a^ | No | No |
| Employment status, n (%) | Yes ^b^ | No | No |
| Anxiety, PROMIS SF v1.0 | Yes ^b^ | No | No |
| Menopause symptoms, MRS | No | Yes | Risk of OSA |
| Acculturation, SL-ASIA | No | No | No |
| Marital status, n (%) | - | Yes ^d^ | - |
| Alcohol consumption | - | No | - |
| Exercise time, n (%) | - | Yes ^e^ | - |
| Sleep quality, PSQI | No | - | - |
| Sleep efficiency, PSQI, n (%) | No | - | - |
| Risk of OSA, Berlin questionnaire, n (%) | Yes ^c^ | - | - |

*Notes*. CVD, Cardiovascular Disease; OSA, Obstructive Sleep Apnea; MRS, Menopausal Rating Scale; SL-ASIA, Suinn-Lew Asian Self-Identity Acculturation; PSQI, Pittsburgh Sleep Quality Index; OSA, Obstructive Sleep Apnea;

^a^ Only in Sleep quality and sleep efficiency model

^b^ All model: Sleep quality, sleep efficiency, risk of OSA, positive with OSA category 1, and subgroup analysis

^c^ Showed direct effect only after adjusted for length of stay in the USA, education, income, numbers of children, employment status, anxiety, menopause symptoms, and acculturation

^d^ Only one category (Single) showed direct effect compared to the reference

^e^ Only one category (Exercise time: 30-60 min / day) showed direct effect compared to the reference

**Table S4** moderating effects of sleep-related characteristics on the relationship between participants characteristics, socioeconomic factors, health-related factors, and cardiovascular disease risk

|  | Dependent variables: CVD risk | | | | | | | | | | | |
| --- | --- | --- | --- | --- | --- | --- | --- | --- | --- | --- | --- | --- |
| Independent variables | Moderators ^b^ | | | | | | | | | | | |
|  | Sleep quality ^a^ | | | | Sleep efficiency ^a^ | | | | Risk of OSA ^a^ | | | |
|  | Coef. | *p* | 95% CI | | Coef. | *p* | 95% CI | | Coef. | *p* | 95% CI | |
| Length of stay in the USA, years | 0.108 | 0.000 | 0.056 | 0.160 | 0.112 | 0.000 | 0.059 | 0.165 | 0.102 | 0.000 | 0.048 | 0.155 |
| Education, n (%) |  |  |  |  |  |  |  |  |  |  |  |  |
| High school equivalent or less |  |  |  |  |  |  |  |  |  |  |  |  |
| Some college or associate degree | -2.740 | 0.046 | -5.431 | -0.048 | -2.558 | 0.070 | -5.331 | 0.215 | -2.511 | 0.074 | -5.275 | 0.253 |
| College degree or higher | -3.397 | 0.002 | -5.571 | -1.223 | -3.281 | 0.005 | -5.525 | -1.036 | -3.674 | 0.001 | -5.907 | -1.441 |
| Household income per year, n (%) |  |  |  |  |  |  |  |  |  |  |  |  |
| < $0 - $30,000 |  |  |  |  |  |  |  |  |  |  |  |  |
| $30,001 - 70,000 | -0.122 | 0.918 | -2.464 | 2.219 | -0.315 | 0.794 | -2.702 | 2.072 | -0.891 | 0.470 | -3.326 | 1.544 |
| > $70,000 | -1.133 | 0.325 | -3.404 | 1.137 | -1.271 | 0.278 | -3.583 | 1.042 | -1.542 | 0.201 | -3.919 | 0.836 |
| Numbers of children, n (%) |  |  |  |  |  |  |  |  |  |  |  |  |
| None |  |  |  |  |  |  |  |  |  |  |  |  |
| 1-2 children | -3.081 | 0.003 | -5.076 | -1.087 | -3.116 | 0.003 | -5.151 | -1.081 | -2.869 | 0.007 | -4.932 | -0.805 |
| Employment status, n (%) |  |  |  |  |  |  |  |  |  |  |  |  |
| Unemployed |  |  |  |  |  |  |  |  |  |  |  |  |
| Employed | -1.717 | 0.018 | -3.136 | -0.299 | -1.787 | 0.016 | -3.240 | -0.335 | -1.900 | 0.011 | -3.360 | -0.440 |
| Anxiety, PROMIS SF v1.0 | 0.108 | 0.023 | 0.016 | 0.201 | 0.117 | 0.017 | 0.022 | 0.212 | 0.171 | 0.001 | 0.076 | 0.266 |
| Menopause symptoms, MRS | -0.083 | 0.337 | -0.253 | 0.087 | -0.145 | 0.079 | -0.308 | 0.017 | -0.180 | 0.025 | -0.337 | -0.023 |
| Acculturation, SL-ASIA | -0.601 | 0.322 | -1.798 | 0.596 | -0.528 | 0.392 | -1.747 | 0.690 | -0.570 | 0.355 | -1.787 | 0.646 |
| Moderator ^b^ | -0.148 | 0.250 | -0.402 | 0.106 | -0.018 | 0.767 | -0.138 | 0.102 | 4.815 | 0.000 | 2.865 | 6.766 |
|  | F( 11, 108) = 5.67, p = 0.000 | | | | F( 11, 108) = 5.57, p = 0.000 | | | | F( 11, 108) = 8.25, p = 0.000 | | | |
| Interaction with moderators | Moderators ^b^ | | | | | | | | | | | |
|  | Sleep quality ^a^ | | | | Sleep efficiency ^a^ | | | | Risk of OSA ^a^ | | | |
|  | Coef. | *p* | 95% CI | | Coef. | *p* | 95% CI | | Coef. | *p* | 95% CI | |
| Length of stay in the USA | 0.006 | 0.551 | -0.014 | 0.027 | -0.005 | 0.238 | -0.014 | 0.004 | -0.117 | 0.129 | -0.269 | 0.035 |
| Education |  |  |  |  |  |  |  |  |  |  |  |  |
| Some college or associate degree | 0.888 | 0.032 | 0.077 | 1.699 | NA |  |  |  |  |  |  |  |
| College degree or higher | 1.069 | 0.001 | 0.454 | 1.684 | -0.210 | 0.124 | -0.479 | 0.059 | 8.493 | 0.000 | 4.000 | 12.986 |
| Numbers of children | 0.069 | 0.890 | -0.918 | 1.056 | 0.026 | 0.896 | -0.363 | 0.415 | 0 |  |  |  |
| Employment status | 0.648 | 0.005 | 0.205 | 1.091 | 0.151 | 0.163 | -0.062 | 0.364 | 1.114 | 0.646 | -3.683 | 5.910 |
| Anxiety | -0.019 | 0.204 | -0.048 | 0.010 | 0.011 | 0.212 | -0.006 | 0.028 | 0.878 | 0.000 | 0.692 | 1.063 |
| Menopause symptoms, MRS | NA |  |  |  | NA |  |  |  | 0.772 | 0.000 | 0.452 | 1.092 |

*Notes*. CVD, Cardiovascular Disease; OSA, Obstructive Sleep Apnea; MRS, Menopausal Rating Scale; SL-ASIA, Suinn-Lew Asian Self-Identity Acculturation; ^a^ Robust regression; ^b^ Moderator

**Table S5** summary of the moderating effects of participants characteristics, socioeconomic factors, health-related factors, and sleep-related characteristics on cardiovascular disease risk

| Independent variables | Dependent variables |
| --- | --- |
|  | CVD risk (n=120) |
|  | Moderator |
| Length of stay in the USA, years | No |
| Education, n (%) | Sleep quality |
| Household income per year, USD, n (%) | No |
| Numbers of children, n (%) | No |
| Employment status, n (%) | Sleep quality |
| Anxiety, PROMIS SF v1.0 | Risk of OSA |
| Menopause symptoms, MRS | Risk of OSA |
| Acculturation, SL-ASIA | No |
| Marital status, n (%) | - |
| Alcohol consumption, n (%) | - |
| Exercise time, n (%) | - |
| Sleep quality, PSQI | - |
| Sleep efficiency, PSQI, n (%) | - |
| Risk of OSA, Berlin questionnaire, n (%) | - |

*Notes*. CVD, Cardiovascular Disease; MRS, Menopausal Rating Scale; SL-ASIA, Suinn-Lew Asian Self-Identity Acculturation; PSQI, Pittsburgh Sleep Quality Index; OSA, Obstructive Sleep Apnea

CVD risk

**Demographic characteristics and**

**Socioeconomic factors:**

- Length of stay in the USA

- Education

- Household income

- Numbers of children

- Employment status

**Health-related factors:**

- Anxiety

- Menopause symptoms

**Figure S1.** Proposed model to estimate paths toward cardiovascular disease risk.

*Notes.* CVD, Cardiovascular Disease

**Sleep-related characteristics:**

- Sleep quality

- Sleep efficiency

- Risk of OSA

**Demographic characteristics,**

**Socioeconomic and health factors:**

- Marital status

- Acculturation

- Alcohol consumption

- Exercise time

**Demographic characteristics and**

**Socioeconomic factors:**

- Length of stay in the USA

- Education

- Household income

- Numbers of children

- Employment status

**Health-related factors:**

- Anxiety

- Menopause symptoms

CVD risk

**Figure S2.** Proposed model to estimate paths toward cardiovascular disease risk (mediation).

*Notes.* CVD, Cardiovascular Disease; OSA, Obstructive Sleep Apnea

**S3A**

CVD risk

- Education

- Employment status

Sleep quality

**S3B**

CVD risk

- Education ^a^

- Anxiety

- Menopause symptoms

Risk of OSA

**Figure S3.** Path diagram for the moderating model of sleep quality (A) and risk of Obstructive Sleep Apnea (OSA) (B). Non-standardized estimates are reported for statistically significant effects shown as solid lines. The effect of sleep quality was significant for education (some college or associate degree: coefficient=0.888, p=0.032; college degree or higher: coefficient=1.069, p=0.001) and employment status (employed: coefficient=0.648, p=0.005). The effect of OSA was significant for anxiety (coefficient=0.878, p<0.001) and menopause symptoms (coefficient=0.772, p<0.001). Other covariates controlled in the models include length of stay in the USA, marital status, household income, number of children, employment status, anxiety, menopause symptoms, and acculturation.

*Notes.* CVD, Cardiovascular Disease; OSA, Obstructive Sleep Apnea

^a^ only college degree
